# Effect of air pollution on the autonomic modulation of heart rate in overweight adults

**DOI:** 10.31744/einstein_journal/2020AO5100

**Published:** 2020-11-25

**Authors:** Luis Henrique Base, Juliana Regis da Costa e Oliveira, Laura Cristina Pereira Maia, Jennifer Yohanna Ferreira de Lima Antão, Celso Ferreira, Celso Ferreira

**Affiliations:** 1 Universidade Federal de São Paulo São PauloSP Brazil Universidade Federal de São Paulo, São Paulo, SP, Brazil.; 2 Faculdade de Medicina do ABC Santo AndréSP Brazil Faculdade de Medicina do ABC, Santo André, SP, Brazil.

**Keywords:** Environmental pollution, Air pollution, Cardiovascular system, Autonomic nervous system, Heart rate, Obesity, Overweight

## Abstract

**Objective:**

To analyze the effect of air pollution on heart rate variability in overweight individuals.

**Methods:**

A total of 46 adult individuals, both sexes, aged between 18 and 49 years and with body mass index >25kg/m^2^ were analyzed. All volunteers were students from public schools of two cities in the state of São Paulo. The clinical, demographic and anthropometric data of each individual, as well as heart rate variability through time domain, geometric and frequency indices were collected. For the air quality analysis, the following variables were investigated: concentration of carbon dioxide, particulate matter, temperature and relative humidity. The analysis was carried out with descriptive and analytical statistics, adopting a level of significance of 5%.

**Results:**

There was a reduction in overall heart rate variability in overweight individuals by the following indices: mean standard deviation of all normal RR intervals, long-term standard deviation of continuous RR intervals, ratio of short-and long-term standard deviation of continuous RR intervals. In addition, the indices responsible for parasympathetic control showed a downward trend in their values, as well as the low frequency index, which represented sympathetic action, although not significant.

**Conclusion:**

Overweight individuals exposed to air pollution had lower heart rate variability than the Control Group.

## INTRODUCTION

Cardiovascular diseases (CVD) is leading cause of death worldwide. In 2017, for example, around 17.8 million of people have died due to the CVD, 8.9 million of have died for ischemic cardiac disease, and 6.2 million have died due to stroke.^(^^1^^)^ In Brazil, CVD are also considered one of the main causes of deaths, and this disease is responsible for approximately 20% of all deaths in the country among individuals older than 30 years of age.^(^^2^^)^

One of the main risk factors for the appearance of diseases is, among other reasons, the overweight.^(^^3^^)^ The World Health Organization (WHO) classifies the level of overweight and obesity by using body mass index (BMI) formula from 25 to 29.9kg/m^2^ and >30kg/m^2^, respectively. Obesity is still a cut-off-point for cardiovascular risk due to the mean abdominal circumference, which is considered of increased risk values of ≥94cm in men and 80cm in women, and substantiated increased risk values of ≥102cm in men and 88cm in women.^(^^4^^)^

Body fat in the abdomen above accepted reference values has been associated with higher incidence of heart diseases, stroke, high blood pressure, dyslipidemias, diabetes, atherosclerosis, coronary heart diseases, among others.^(^^5^^)^

Other risk factor for the CVD is air pollution that is considered the 10th leading global risk factor for death. Pollution consists of suspended particles in the air, known as particle matter (PM), responsible for the majority of adverse effects.^(^^6^^)^ A study on the relationship between air pollution and effects on the cardiovascular system showed that per 10µg/m^3^ increase of PM with diameter lower than 10µm (PM_10_) and PM with diameter lower than 2.5µm (PM_2,5_), caused an increase in hospitalization and cardiovascular death indices.^(^^7^^)^ This fact can be explained due to number of physiological changes that occurred in cardiovascular system, such as inflammation, coagulation, and abnormal heart rhythm.^(^^8^^,^^9^^)^

Little cardiac changes can be detected by the autonomic nervous system (ANS), which influences the cardiovascular system controlled by parasympathetic and sympathetic routes, by using a quite common tool, heart rate variability (HRV).^(^^8^^)^

This method is able to characterize and detect a number of morbid conditions, therefore suggesting that HRV can be the major cause of loss of homeostasis, and may contribute for early diagnosis.^(^^8^^)^

To understand effect of air pollution in autonomic modulation of heart rate of overweight adults is needed as way to determine the marker of cardiovascular prognosis as well as to establish reference values to improve therapeutic intervention.

## OBJECTIVE

To analyze the effect of air pollution in autonomic modulation of heart rate variability in overweight individuals.

## METHODS

### Selected areas

This was a cross-sectional study, submitted and approved to the Research Ethics Committee of the *Universidade Federal de São Paulo,* and registered under the protocol number 1,113.059 (CAAE: 45729015.4.0000.5505), according to the resolution 466/12 of December 12th, 2012, from the National Health Council. All procedures of the study were explained to participants as well as the objective of the study, data collection parameters, potential risks and benefits of their participation. Those who agreed to participate signed the consent form.

The contaminated area, the municipality of Cubatao, was determined based on studies conducted by the Environmental Company of the State of São Paulo (CETESB),^(^^10^^,^^11^^)^ which identified toxic substance in the area. The air quality index of in state of Sao Paulo^(^^12^^)^ showed high indices of air population in Cubatao. The control area, the municipality of Peruibe, was chosen because the city is the most distant one from the experimental area within Baixada Santista (coastal region) and because there are no evidences of high air population index in that region according to the CETESB.

### Participants of the study

All volunteers were students living at selected areas of both sexes from youth and adult formal education programs of public schools from each municipality. Their BMI was >25kg/m^2^, and age range between 18 and 49 years. Exclusion criteria were existence of any disease associated with the use of continuous medication that potently affected cardiovascular system, an error greater than 5%in HRV data, and those with extreme values (outliers) in the studied variables. The study was conducted between March and June 2016, and included all individuals who were interested in collaborate with the study (convenience sampling).

### Assessment measures

First, we collected personal data such as sex, age, formal education, smoking habit, pre-existing condition, and continuous use of medication, and also anthropometric features such as weight, height, abdominal circumference and BMI. Weight was determined using a digital scale (Marte^®^, Sao Paulo, Brazil) with maximal capacity of 150kg and precision of 100g. Height was determined using a portable stadiometer (Alturexata^®^, Sao Paulo, Brazil), made of wood, of 2.13m, subdivided in centimeters and millimeters. Abdominal circumference was obtained in lowest angle between lower ribs and iliac crest by using an inelastic metric band. BMI was calculated using the formula^(^^13^^)^ weight/height^2^, weight in kilograms by height in meters. The systolic blood pressure (SBP), the diastolic blood pressure (DBP), and heart rate (HR) were also registered.

We also measured the degree of physical activity by using the International Physical Activity Questionnaire (IPAQ), which was classified as high, medium, low level of physical activity.^(^^14^^)^

### Heart rate variability

HRV was conducted in a room under stable temperature (21 to 23°C) and humidity (40% to 60%) at night between 7 p.m. and 9 p.m. to standardize effects of circadian rhythm. Individuals were placed in a sitting position with spontaneous breathing for 20 minutes. Chest strap as well as heart monitor were placed on their chest aligned to distal third of sternum and a wrist based heart rate monitor.

Data were registered on Polar RS800CX watch and transferred to the computer to be analyzed by the RS800CX Polar watch software. We used thousands of intervals within consecutive RR intervals. After, manual filtering was conducted using a spreadsheet in Microsoft Excel to exclude premature and artifacts and ectopic beats. We selected only series with more than 95% of regular beats.^(^^8^^,^^15^^,^^16^^)^

The software used for HRV assessment was the Kubios Heart Rate Variability (Kubios HRV) that analysis linear indices, time domain, frequency, and geometric indices.^(^^8^^,^^15^^,^^16^^)^ In time domain, we considered the root mean square of successive differences between normal heartbeats (rMSSD), expressed in milliseconds, percentage of successive differences of RR interval in which absolute value exceed 50ms (pNN50), and standard deviation of the normal-to-normal RR intervals (SDNN) expressed in milliseconds. Among geometric indices, we considered triangular index (RRtri), triangular interpolation of NN interval histogram (TINN) and Poincaré plot composed by standard deviation of instant heart rate variability and short term heart rate (SD1), long term standard deviation of continuous RR intervals (SD2) and their both relationships (SD1/SD2) showing a ratio between short and long term variations of RR intervals.

In frequency domain, we used spectral component of low frequency (LF), ranging between 0.04 to 0.15Hertz, expressed in ms^2^ and absolute units (nu); high frequency (HF), ranging from 0.15 to 0.4 Hertz, expressed in m^2^ and nu; and ratio between these components LF/HF.^(^^8^^,^^15^^,^^16^^)^

### Air quality

Portable devices used were AZ Instrument 77535 CO_2_/Temp./RH measurer to measurement of temperature, relative humidity (RH) and carbon dioxin concentration, and portable counter of HHP particles + Met One Instruments to measure MP concentration from 0.3 to 3 μm.

The equipment were placed to 1.5m above the ground to obtain reliable measure. After, the data obtained in measure points were analyzed and its mean values were calculated.

To determine air quality, we conducted measures in reference site in each municipality (reference standard values). In Cubatao, we selected the public square named “Praça emancipadores” where the city hall is located. In Peruibe we chosen the Monsenhor Lino dos Passos Square, where the church of the city is located. In addition, air quality was also measured at both public schools within classrooms (internal environmental) and in schoolyard (external environmental). Air quality measure in internal/external environmental was conducted at night, whereas collection at reference locals was conducted in the afternoon between 1 p.m. and 2 p.m.

### Statistical analysis

All statistical analyses were conducted using the R software, version 3.5.1, and, for all tests, we fixed in 5% (p<0.05) the level of rejection of the null hypothesis. Results were expressed on means and standard deviations for data with normal distribution, and median and interquartile intervals for those with non-normal distribution.

We applied Shapiro-wilk test to assess normal distributions, as well as log and square root transformation, when necessary. To compare statistics between groups of categorical variables, we used the Person's χ^2^ test and the Fisher´s exact test, for numerical variables we used the Mann-Whitney test and Student *t* test.

## RESULTS

### General characteristics of population

**Figure 1:**
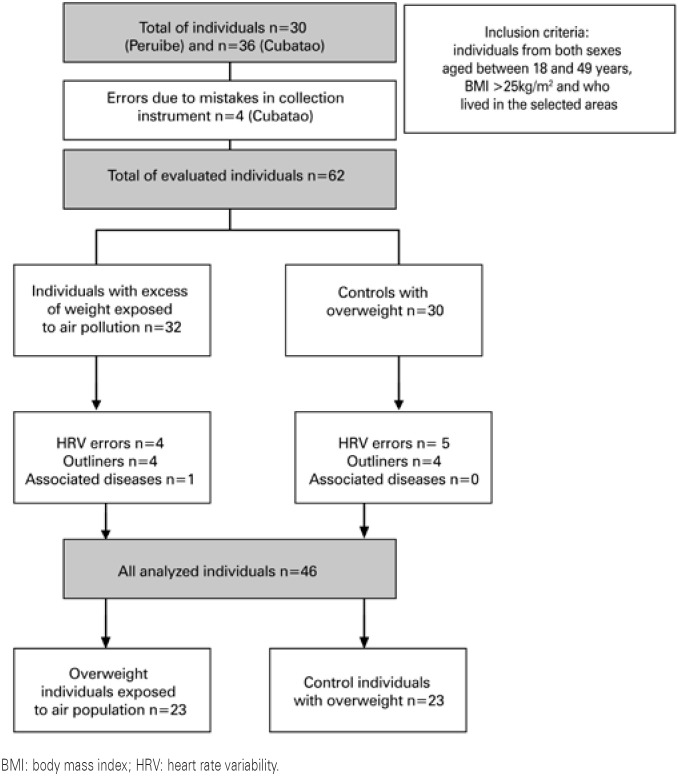
Steps to select participants for the study

First, 66 individuals were identified, of these four had mistakes in collected instrument (heart rate). Of the 62 evaluated individuals, 46 were included in the final analysis and composed the Control Group formed by controls overweight individuals (n=23), and Experimental Group that included overweight individuals exposed to air pollution (n=23), figure 1 .

Two analyzed groups did not show differences in anthropometric features, demographic and clinical data. Individuals’ mean age were 36 and 41 years in Experimental Group and Control Group, respectively. Of these 13 (56.5% of population) were women in Experimental Group and 13 (56.5%) were men in Control Group. In both groups, 69.6% of individuals were overweight with mean BMI of 27.6kg/m^2^ and 28.6kg/m^2^ in Control Group and Experimental Group, respectively. Anthropometric features, demographic data, and clinical variables of studied population are shown in table 1 .

**Table 1:** Characterization and comparison of general characteristics of individuals living in the municipalities of Cubatao and Peruibe

Variables		CG	EG	p value
		(n=23)	(n=23)	
Age, years		36 (25-40.5)	41 (29-45.5)	0.187 *
Formal education, years		5 (4.5-9.5)	6 (2.5-8)	0.635 *
Body weight, kg		77.5 (69.7-82.4)	76.4 (69-83.4)	0.766 *
BMI, kg/m^2^		27.6 (26.8-31.1)	28.6 (26.4-30.7)	0.877 *
HR, bpm		73 (66-78.5)	72 (63.5-81.5)	0.652 *
SBP, mmHg		120 (110-130)	120 (120-130)	0.229 *
DBP, mmHg		80 (80-82.5)	80 (75-85)	0.858 *
Height, m		1.64±0.09	1.63±0.09	0.617 †
Abdominal circumference, cm		90±9.21	93.8±9.51	0.182 †
Sex				0.555 ‡
	Men	56.5 (13)	43.4 (10)	
	Women	43.4 (10)	56.5 (13)	
IPAQ				0.732 §
	Low	4.4 (1)	30.4 (7)	
	Mean	65.2 (15)	60.9 (14)	
	High	30.4 (7)	8.7 (2)	
Smoking				0.108 §
	No	100 (23)	73.9 (17)	
	Yes	0 (0)	26.1 (6)	
Classification of BMI				0.571 §
	Underweight (<18.5kg/m^2^)	0 (0)	0 (0)	
	Normal weight (18.5>24.9kg/m^2^)	0 (0)	0 (0)	
	Overweight (25>29,9kg/m^2^)	69.6 (16)	69.6 (16)	
	Obesity (≥30kg/m^2^)	30.4 (7)	30.4 (7)	

Results expressed by median and interquartile range (Q1 and Q3).

* Mann-Whitney test;

† student *t* test;

‡ Pearson's χ^2^ test;

§ Fisher's exact test.

CG: Control Group; EG: Experimental Group; BMI: body mass index; HR: heart rate; SBP: systolic blood pressure; DBP: diastolic blood pressure; IPAQ: International Physical Activity Questionnaire.

### Characteristics of air quality

In relation to characteristics of external environmental, according to reference standard deviation in the municipality of Cubatao and Peruibe, all items were statistically higher in Experimental Group compared with Control Group, table 2 .

**Table 2:** Comparison characteristics of external environmental mean (reference value) in the municipalities of Cubatao and Peruibe

Variables		Mean/median	CI/IQR		p value
CO_2_, ppm					<0.001 *
	CG	454.5	450.7	458.2	
	EG	479.1	473.1	485.1	
Temperature, °C					<0.001 *
	CG	25.3	24.0	26.6	
	EG	20.2	19.5	20.9	
Humidity, %					0.003 †
	CG	46.4	44.9	46.7	
	EG	77.6	75.1	78.4	
PM 0.3 (x10^6^)					0.001 †
	CG	59.5	59.5	62.7	
	EG	214.7	211.4	216.8	
PM 0.5 (x10^6^)					0.001 †
	CG	4.9	4.7	5.4	
	EG	92.3	89.5	93.2	
PM 1.0 (x10^6^)					0.001 †
	CG	1.1	1.0	1.1	
	EG	11.6	10.2	12.0	
PM 3,0 (x10^6^)					0.001 *
	CG	0.9	0.9	0.9	
	EG	2.3	1.8	2.4	

* parametric data (median and CI);

† non-parametrical data (median and IQR).

CI: confidence interval; IQR: interquartile interval (Q1 and Q3); CO_2_: carbon dioxide; CG: Control Group; EG: Experimental Group; PM: Particulate matter.

In the internal environmental of school (classroom), only carbon dioxide items and temperature were significantly with higher values in Experimental Group compared with Control Group ( Table 3 ).

**Table 3:** Comparison of characteristics of internal environmental of school, according to numerical variables in municipalities of Cubatao and Peruibe

Variables		Mean	IQR (Q1 – Q3)		p value
CO_2_, ppm					<0.001
	CG	528.0	520.7	542.2	
	EG	569.0	546.0	683.0	
Temperature, ° C					<0.001
	CG	18.0	18.0	19.0	
	EG	19.0	19.0	20.0	
Humidity, %					0.409
	CG	73.9	72.2	75.6	
	EG	85.6	69.6	87.3	
PM 0,3 (x10^6^)					0.724
	CG	129.6	59.7	136.9	
	EG	237.3	32.2	252.6	
PM 0.5 (x10^6^)					0.852
	CG	7.9	4.1	8.6	
	EG	79.5	1.9	92.1	
PM 1.0 (x10^6^)					0.852
	CG	1.1	1.0	1.2	
	EG	8.1	0.3	9.7	
PM 3.0 (x10^6^)					0.851
	CG	0.8	0.8	0.9	
	EG	2.0	0.2	2.3	

Mann-Whitney test.

IQR: interquartile range; CO_2_: carbon dioxide; CG: Control Group; EG: Experimental Group; PM: particulate matter.

In the characterization of external environmental of school (schoolyard) carbon dioxide and humidity were significant with higher values in Experimental Group compared with Control Group ( Table 4 ).

**Table 4:** Comparison of characteristics of external environmental (schoolyard) of the school according to numerical variable in the municipalities of Cubatao and Peruibe

Variables		Mean/median	CI/IQR		p value
CO_2_, ppm					0.005 *
	CG	471.0	465.0	476.5	
	EG	539.0	466.0	554.0	
Temperature, ° C					0.295 *
	CG	17.0	16.0	17.0	
	EG	17.0	16.0	17.0	
Humidity, %					0.001 †
	CG	82.9	80.9	84.8	
	EG	88.4	85.6	91.2	
PM 0.3 (x10^6^)					0.221 *
	CG	127.2	66.1	161.8	
	EG	101.9	46.7	220.7	
PM 0.5 (x10^6^)					0.645 *
	CG	7.7	5.0	12.1	
	EG	8.5	2.6	111.7	
PM 1.0 (x10^6^)					0.701 *
	CG	1.1	1.0	1.5	
	EG	0.5	0.4	15.5	
PM 3.0 (x10^6^)					0.352 *
	CG	0.8	0.8	0.9	
	EG	0.5	0.3	3.8	

* non-parametric data (median and IQR);

† parametric data (mean and CI).

CI: confidence interval; IQR: interquartile range; CO_2_: carbon dioxide; CG: Control Group; EG: Experimental Group; PM: particulate matter.

### Heart rate variability

The items SDNN, SD2, RRTri and TInn that represented modulation of general HRV showed significance after analytical statistics ( Table 5 ). In general, a reduction of these variables occurred in Experimental Group compared with Control Group.

**Table 5:** Linear indices, time domain and frequency, and geometric indices of heart rate variability of selected groups

HRV indices	CG	EG	p value
	(n=23)	(n=23)	
	Mean (CI)	Mean (CI)	
SDNN, ms	48.2 (43.2-53.1)	38.7 (33.5-43.8)	0.012 *
rMSSD, ms	27.5 (23.5-31.4)	22.6 (19.3-25.8)	0.061 *
pNN50, %	7.9 (4.4-11.4)	4.7 (2.9-6.6)	0.079 †
LFms^2^	738.1 (601.8-874.3)	541.8 (403-680.5)	0.054 *
LFnu	73.8 (69-78.5)	71.6 (67.1-76)	0.492 *
HFms^2^	254.8 (183.9-325.6)	208 (145.2-270.7)	0.441 ‡
HFnu	26 (21.2-30.7)	28.3 (23.8-32.7)	0.489 *
LF/HF	3.6 (2.7-4.5)	3.1 (2.3-3.9)	0.460 ‡
SD1, ms	19.5 (16.7-22.2)	16 (13.7-18.2)	0.062 *
SD2, ms	65.1 (58.2-71.9)	52.1 (44.9-59.2)	0.013 *
SD1/SD2, ms	0.304 (0.267-0.340)	0.317 (0.279-0.354)	0.625 *
RRTri, ms	12.9 (11.6-14.1)	10.3 (8.9-11.6)	0.009 *
TINN, ms	214.3 (193.8-234.7)	172.8 (150.5-195)	0.010 *

* student *t* test without adjust;

† student *t* test after adjust with squared root;

‡ student *t* test after adjusted log transformation.

HRV: heart rate variability; CG: Control Group; EG: Experimental Group; IC: confidence interval; SDNN: standard deviation of the normal-to-normal RR intervals; rMSSD: root mean square of successive differences between normal heartbeats; pNN50: percentage of successive differences of RR interval in which absolute value exceed 50ms; FL: component of low frequency; nu: normalized unit; HF: high frequency component; SD1: standard deviation from instant hear rate variability to short-term heart rate; SD2: standard deviation to long-term of interval of continuous RR; SD1/SD2 relation: dispersion of perpendicular score to line of identify dispersion of score to identify of line; RRTri: triangle index; TINN: triangle interpolation of histogram of NN intervals.

## DISCUSSION

Results of the present study showed that Experimental Group composed by overweight individuals exposed to air pollution presented reduction in values of SNDD indices, SD2, RRTri and TINN (which corresponded to global modulation of HRV) compared with Control Group, suggesting that in these individuals HRV was reduced.

Publish literature suggests standardization of indices values of HRV among possible healthy individuals. An attempt was conducted in the study by Sammito et al.,^(^^17^^)^ that registered HRV values long duration (24 hours) with use of electrocardiogram (ECG) in healthy adults. As a result, authors observed differences in values among different ages and sexes. Among variables of time domain, mean values of SDNN and rMSSD variables for men aged 30 to 40 years were 48.98 and 40.71millisecond, respectively. In relation to pNN50 variable for the same age range was 13.23%. Among women from 30 to 40 years, values of means were lower: 42.39 for SDNN, 36.50 for rMSSD, and 11.43% for pNN50. In the same study,^(^^17^^)^ frequency domain indices were also studied. Mean values of FL and HF variables in normalized units (FLnu and HFnu) for men aged 30 to 40 years were 75.46 and 24.54, respectively. Among men within this age range, the FL/HF was 3.08. Women aged 30 to 40 years presented mean of 67.81 for LFnu, 32.19 for HFnu, and 2.11 for FL/HF.^(^^17^^)^

Other study, conducted by the Sandercock et al.,^(^^18^^)^ we also measured values of HRV in healthy adults, however, with short-term recording (5 to 10 minutes) with the use of Polar heart rate. Values of indices from HRV range according to intensity of physical exercise. As a result values found between men and women with BMI between 22 and 23kg/m^2^, and mean age of 23 years (ranging from 18 and 33 years), as low, moderate, and high level of physical exercise were 55, 75 and 64 millisecond for SDNN. In addition, 56, 78, 60 millisecond for rMSSD; 58, 57 and 65 for LFnu; 42, 43 and 35 for HFnu; 6.95, 7.26 and 7.31ms^2^ for LFms^2^; 6.59, 6.95 and 6.60 millisecond to square for HFms^2^; and 2.0, 1.7, and 2.6 for LF/HF, respectively.^(^^18^^)^

In general, values of SDNN, rMSSD and pNN50 indices of cited studies (healthy adults) were higher compared with values of control and Experimental Group of this study; however, the index domain frequency ranged according to sex and level of physical exercise, suggesting that this weight gain can be associated with reduction of HRV, indicating autonomic dyscontrol.^(^^19^^)^ These finding corroborated with other mentioned studies in published literature, which compared overweight individuals and normal weight.^(^^20^^–^^26^^)^

One of the possible explanations on these changes in the HRV is activation of baroreflex. A study^(^^27^^)^ that tested hypothesis from baroreflex sensitivity (BRS), evaluated by the indirect measure of aortic pressure, was related with obesity, selected on the group including 30 women with BMI of 30kg/m^2^ and group of 30 controls with BMI of 25kg/m^2^. The HRV was estimated by the activity from cardiac ANS by the HRV. In this way, BRS was lower in obese women, already values of HF and LF were greater in thin participants from obese (1,079.2 *versus* 224.1ms^2^, and 411.8 *versus* 235.8ms^2^, respectively) and value from LF/HF was higher obese individuals (0.82 *versus* 0.57). The BMI, age and activity from parasympathetic nervous system are the main determining from BRS. Baroreflex behavior is of clinical relevance, because when attenuated, represented negative prognostic factor in CVD, which is common in obesity.^(^^27^^)^

When individuals with overweight exposed to air pollutions, the HRV values showed even more changed. In general, the literature point outs to the significant inverse relation between exposition to atmospheric pollutions, mainly to MP, an reduction of SDNN, rMSSD, HF indices, even in FL for overweight individuals exposed to high levels of these air particles.^(^^28^^–^^34^^)^ However, in the result of this study, only the SDNN indices in the domain of time, and indices SD2, RRTri and TINN of geometric indices presented significantly reduction than Control Group, therefore, suggesting changes in global variability. Other indices, such as rMSSD, pNN50, LFms^2^, and SD1, also showed lower values than Control Group, although they were not statistically significant, ranging between 0.054 and 0.07.

### Limitation

Limitations of this study included some measurements that could be not done, for example, the use of waist-hip ratio, the analysis of risk of development of CVD, which would be better divided between groups. Other limitation was sample loss – around 20 exclusions (30.3% of sample loss) – mainly due to errors in HRV. Therefore, we recommend adding 31% in sample calculation in future studies. In addition, we could not conduct collection of other pollutions in which would show, more precisely, the expose of different pollutants in studied individuals.

## CONCLUSION

Overweight individuals exposed to air population showed lower variability of global cardiac frequency than Control Group. In addition, parasympathetic and sympathetic modulation showed tendency of declining, although not significant. Our findings suggest that overweight individuals exposed to air pollution present autonomic imbalance and, therefore, a greater susceptibility to the development of cardiovascular diseases.
