# Supplementary material for: Effect of air pollution on the autonomic modulation of heart rate in overweight adults
Source: Einstein (Sao Paulo). 2020 Nov 25;18:eAO5100. doi: 10.31744/einstein_journal/2020AO5100 (PMC7690934; doi:10.31744/einstein_journal/2020AO5100)
Supplement: Translation

# Translation: Efeito da poluição atmosférica na modulação autonômica da frequência cardíaca em indivíduos com excesso de peso

**Authors:** Luis Henrique Base, Juliana Regis da Costa e Oliveira, Laura Cristina Pereira Maia, Jennifer Yohanna Ferreira de Lima Antão, Celso Ferreira, Celso Ferreira

**Affiliations:** 1 São PauloSP Brasil Universidade Federal de São Paulo, São Paulo, SP, Brasil.; 2 Santo AndréSP Brasil Faculdade de Medicina do ABC, Santo André, SP, Brasil.

**Keywords:** Poluição ambiental, Poluição do ar, Sistema cardiovascular, Sistema nervoso autônomo, Frequência cardíaca, Obesidade, Sobrepeso

## Abstract

**Objetivo:**

Analisar o efeito da poluição atmosférica na variabilidade da frequência cardíaca em indivíduos com excesso de peso.

**Métodos:**

Foram analisados 46 indivíduos adultos, de ambos os sexos, com idade entre 18 e 49 anos e índice de massa corporal >25kg/m^2^. Todos os voluntários eram estudantes da Educação de Jovens e Adultos em duas cidades do estado de São Paulo. Foram coletadas informações clínicas, demográficas e antropométricas de cada indivíduo, bem como a variabilidade da frequência cardíaca, por meio dos índices do domínio do tempo, geométricos e de frequência. Para a análise da qualidade do ar, foram investigadas as variáveis concentração de dióxido de carbono, material particulado, temperatura e umidade do ar. A análise se deu por estatística descritiva e analítica, adotando-se nível de significância de 5%.

**Resultados:**

Ocorreu redução na variabilidade da frequência cardíaca geral nos indivíduos com excesso de peso por meio dos índices geométricos (desvio padrão a longo prazo dos intervalos RR contínuos, e a razão entre as variações curta e longa dos intervalos RR) e o domínio do tempo (desvio padrão da média de todos os intervalos RR normais). Índices responsáveis pelo controle parassimpático mostraram-se com tendência de queda em seus valores, bem como o índice de baixa frequência que representava a ação do simpático, embora com valores não significativos.

**Conclusão:**

Indivíduos com excesso de peso expostos à poluição atmosférica apresentaram menor variabilidade da frequência cardíaca em comparação ao Grupo Controle.

## INTRODUÇÃO

As doenças cardiovasculares (DCV) estão dentre as principais causas de mortes no mundo. Em 2017, por exemplo, cerca de 17,8 milhões de pessoas morreram por DCV, sendo 8,9 milhões de mortes por doença cardíaca isquêmica e 6,2 milhões de mortes por acidente vascular cerebral (AVC).^(^^1^^)^No Brasil, as DCV são também consideradas uma das principais causas de morte, atingindo aproximadamente 20% de todas as mortes brasileiras em indivíduos acima de 30 anos de idade.^(^^2^^)^

Um dos fatores de risco para surgimento dessas doenças é, entre outros, o excesso de peso.^(^^3^^)^A Organização Mundial da Saúde (OMS) classifica os níveis de sobrepeso e obesidade, por intermédio da fórmula do índice de massa corporal (IMC) em 25 a 29,9kg/m^2^e >30kg/m^2^, respectivamente. A obesidade ainda estabelece um ponto de corte para o risco cardiovascular por meio da medida de circunferência abdominal, considerando risco aumentado valor ≥94cm em homens e 80cm em mulheres, e risco aumentado substanciado para ≥102cm em homens e 88cm em mulheres.^(^^4^^)^

A gordura corporal na região abdominal acima dos valores aceitáveis de referência tem sido associada à maior incidência de cardiopatias, AVC, hipertensão arterial, dislipidemias, diabetes, aterosclerose, doenças coronarianas, entre outras.^(^^5^^)^

Outro fator de risco para as DCV é a poluição atmosférica, considerada o décimo principal fator de risco de morte mundial. Ela consiste em partículas suspensas no ar, conhecidas como material particulado (MP), responsáveis pela maioria desses efeitos adversos.^(^^6^^)^Investigação sobre a relação entre poluição do ar e efeitos no sistema cardiovascular demonstrou que a cada aumento de 10µg/m^3^de MP com diâmetro menor que 10µm (MP_10_) e MP com diâmetro menor que 2,5µm (MP_2,5_) ocorreu aumento nos índices de hospitalização e mortalidade cardiovascular.^(^^7^^)^Esse fato pode ser explicado devido a uma série de modificações fisiopatológicas que ocorre no sistema cardiovascular, envolvendo inflamação, coagulação e ritmo cardíaco.^(^^8^^,^^9^^)^

Assim, pequenas alterações cardíacas podem ser detectadas por meio do sistema nervoso autônomo (SNA), que influencia no controle do sistema cardiovascular, por intermédio das vias parassimpática e simpática, usando uma ferramenta bastante utilizada: a variabilidade da frequência cardíaca (VFC).^(^^8^^)^

Esse método é capaz de caracterizar e detectar uma série de condições mórbidas, sugerindo que a VFC possa ser um marcador da perda da homeostase, contribuindo, assim, para diagnósticos mais precoces.^(^^8^^)^

Faz-se, então, necessária a compreensão do efeito da poluição atmosférica na modulação autonômica da frequência cardíaca de adultos com excesso de peso, como forma de identificar um marcador de prognóstico cardiovascular, bem como valores de referência para melhor intervenção terapêutica.

## OBJETIVO

Analisar o efeito da poluição atmosférica na modulação autonômica da frequência cardíaca em indivíduos com excesso de peso.

## MÉTODOS

### Áreas selecionadas

Estudo transversal, submetido e aprovado pelo Comitê de Ética em Pesquisa da Universidade Federal de São Paulo, e registrado com o número de protocolo 1.113.059 e CAAE: 45729015.4.0000.5505, obedecendo à resolução 466/12 do Conselho Nacional de Saúde de 12 de dezembro de 2012. Todos os procedimentos adotados foram explicados aos participantes, como os objetivos do estudo e os parâmetros coletados, bem como possíveis riscos e benefícios de sua participação. Aqueles que concordaram em participar assinaram o Termo de Consentimento Livre e Esclarecido (TCLE).

A área contaminada, município de Cubatão, foi determinada baseando-se nos estudos realizados pela Companhia Ambiental do Estado de São Paulo (CETESB),^(^^10^^,^^11^^)^que comprovou fontes de substâncias tóxicas nesse território. O relatório de qualidade do ar no estado de São Paulo^(^^12^^)^mostrou altos índices de poluição de ar no município. A área controle, o município de Peruíbe, foi escolhida por ser a cidade mais distante em relação à área experimental dentro da Baixada Santista e por não existirem evidências de altos níveis de poluição atmosférica na região segundo a CETESB.

### Sujeitos do estudo

Todos os voluntários eram estudantes da Educação de Jovens e Adultos (EJA) de escolas municipais e/ou estaduais de cada município, de ambos os sexos, com IMC >25kg/m^2^, idade entre 18 e 49 anos, e que residiam nas áreas selecionadas. Os critérios de exclusão considerados foram: qualquer patologia associada ou uso de medicamento contínuo que interferissem no sistema cardíaco, erro maior que 5% nos dados da VFC e aqueles com valores extremos ( *outlines* ) nas variáveis estudadas.

O estudo foi realizado entre os meses de março e junho de 2016, contando com a participação de todos que se interessaram em colaborar com a pesquisa (amostra de conveniência).

### Medidas de avaliação

Primeiramente, foram coletados dados pessoais, como sexo, idade, escolaridade, ato de fumar, doença existente e uso de medicamento contínuo, e antropométricos, como peso, altura, circunferência abdominal e IMC. O peso foi determinado por balança digital (Marte^®^, São Paulo, Brasil), com capacidade até 150kg e subdivisões até 100g. A altura foi determinada utilizando estadiômetro (Alturexata^®^, São Paulo, Brasil) em madeira, de 2,13m, com subdivisões em centímetros e milímetros. A circunferência abdominal foi obtida no menor ângulo entre as últimas costelas e a crista ilíaca, por meio de fita métrica inelástica. O IMC foi calculado pela fórmula:^(^^13^^)^peso/altura^2^, com o peso em quilogramas e a altura em metros. A pressão arterial sistólica (PAS), a pressão arterial diastólica (PAD) e frequência cardíaca (FC) foram também registradas.

Também foi checado o grau de atividade física por intermédio do questionário *International Physical Activity Questionnaire* (IPAQ), que o classificou como alto, médio e baixo nível de atividade física.^(^^14^^)^

### Variabilidade da frequência cardíaca

A VFC foi realizada em uma sala sob circunstâncias estáveis de temperatura (21 a 23°C) e de umidade (40% a 60%), no período noturno entre às 19h e 21h, para padronizar os efeitos do ritmo circadiano. Os indivíduos foram posicionados na postura sentada com respiração espontânea durante 20 minutos. A cinta bem como o monitor cardíaco foram posicionados no tórax, alinhado com o terço distal do esterno e o receptor de FC no punho do indivíduo.

Os dados foram registrados no relógio Polar RS800CX e transferidos para o computador, para serem analisados pelo software *Polar Precision Performance* . Foram utilizados mil intervalos dentro dos intervalos RR consecutivos. Após, foi realizada filtragem manual, por meio do programa Microsoft Excel, para eliminação de batimentos ectópicos prematuros e artefatos. Foram selecionados somente séries com mais de 95% de batimentos regulares.^(^^8^^,^^15^^,^^16^^)^

O software utilizado para a avaliação da VFC foi o Kubios Heart Rate Variability (Kubios HRV), por meio da análise dos índices lineares, domínio do tempo e da frequência, e dos índices geométricos.^(^^8^^,^^15^^,^^16^^)^No domínio do tempo, foram considerados os índices raiz quadrada da média do quadrado das diferenças entre intervalos RR normais adjacentes (rMSSD), expressa em milissegundos; percentagem das diferenças sucessivas do intervalo RR cujo valor absoluto excedeu 50ms (pNN50); e desvio padrão de todos os intervalos RR normais (SDNN) expresso em milissegundos. Entre os índices geométricos, foram considerados: índice triangular (RRtri), interpolação triangular de histograma de intervalos NN (TINN) e *plot* de Poincaré composto por desvio padrão da variabilidade instantânea batimento a batimento em curto prazo (SD1), desvio padrão a longo prazo dos intervalos RR contínuos (SD2) e relação de ambos (SD1/SD2) mostra a razão entre as variações curta e longa dos intervalos RR. No domínio da frequência, foram utilizados os componentes espectrais de baixa frequência (LF), na faixa entre 0,04 a 0,15Hertz, expressa em ms^2^e unidades absolutas (nu); de alta frequência (HF), na faixa de variação de 0,15 a 0,4Hertz, expressa em ms^2^e nu; e a razão entre estes componentes LF/HF.^(^^8^^,^^15^^,^^16^^)^

### Qualidade do ar

Os dispositivos portáteis utilizados foram o AZ Instrument 77535 Medidor de CO_2_/Temp./RH, para medir a temperatura, a umidade relativa (UR) e as concentrações de dióxido de carbono, e contador portátil de partículas HHP + da *Met One Instruments* , para medir a concentração de MP de 0,3 a 3μm. Os equipamentos foram colocados a 1,5m acima do solo, para obter uma medição confiável. Em seguida, os dados obtidos nos pontos de medição foram analisados e seus valores médios, calculados.

Para determinar a qualidade do ar, foram realizadas medições em locais de referência em cada município (valores de referência padrão). Em Cubatão, foi selecionada a Praça dos Emancipadores, onde fica a prefeitura, e, em Peruíbe, foi incluída a Praça Monsenhor Lino dos Passos, onde fica a paróquia da cidade, além da coleta de dados da qualidade do ar dentro da sala de aula (ambiente interno da coleta) e no pátio da escola (ambiente externo) também em ambos os municípios. As coletas da qualidade do ar em ambiente interno/externo foram realizadas no período noturno, enquanto a coleta nos locais de referência foi feita no período vespertino entre 13h e 14h.

### Análise estatística

Todas as análises estatísticas foram conduzidas usando o software R, versão 3.5.1, e, para todos os testes, fixou-se em 5% (p<0,05) o nível para rejeição da hipótese de nulidade. Os resultados foram expressos em média e desvio padrão para dados com distribuição normal, e mediana e intervalo interquartil para aqueles com distribuição não normal.

Aplicou-se o teste de Shapiro-Wilk para avaliar a normalidade das distribuições, bem como a transformação da raiz quadrada e logarítmica, quando necessário.

Para comparação entre os grupos das variáveis categóricas, foram utilizados o teste χ^2^de Person e teste exato de Fisher, e, para as variáveis numéricas, foram utilizados o teste de Mann-Whitney e o teste *t* de Student.

## RESULTADOS

### Características gerais da população

Inicialmente, 66 indivíduos foram identificados, dos quais quatro apresentaram erros no instrumento de coleta (cardiofrequencímetro). Dos 62 indivíduos analisados, 46 foram incluídos nas análises finais e compuseram os Grupos Controle, formado por indivíduos controles com excesso de peso (n=23), e Experimental, de indivíduos com excesso de peso expostos à poluição atmosférica (n=23), como visto na figura 1 .

**Figura 1:** Etapas de seleção dos indivíduos do estudo

Os dois grupos analisados não mostraram diferenças em relação aos dados antropométricos, demográficos e clínicos. As medianas de idade dos indivíduos foram de 36 e 41 anos para os Grupos Experimental e Controle, respectivamente, sendo 13 indivíduos (56,5% da população) do sexo feminino no Grupo Experimental e 13 indivíduos (56,5% da população) do masculino para o Grupo Controle. Em ambos os grupos, 69,6% dos indivíduos apresentaram IMC indicativo de sobrepeso, com mediana de 27,6kg/m^2^e 28,6kg/m^2^para os Grupos Controle e Experimental, respectivamente. As variáveis antropométricas, demográficas e clínicas da população estudada estão dispostas na tabela 1 .

**Tabela 1:** Caracterização e comparação das características gerais dos indivíduos residentes nos municípios de Cubatão e Peruíbe

Variáveis		GC	GE	Valor de p
		(n=23)	(n=23)	
Idade, anos		36 (25-40,5)	41 (29-45,5)	0,187 *
Escolaridade, anos		5 (4,5-9,5)	6 (2,5-8)	0,635 *
Peso corporal, kg		77,5 (69,7-82,4)	76,4 (69-83,4)	0,766 *
IMC, kg/m^2^		27,6 (26,8-31,1)	28,6 (26,4-30,7)	0,877 *
FC, bpm		73 (66-78,5)	72 (63,5-81,5)	0,652 *
PAS, mmHg		120 (110-130)	120 (120-130)	0,229 *
PAD, mmHg		80 (80-82,5)	80 (75-85)	0,858 *
Altura, m		1,64±0,09	1,63±0,09	0,617 †
Circunferência abdominal, cm		90±9,21	93,8±9,51	0,182 †
Sexo				0,555 ‡
	Masculino	56,5 (13)	43,4 (10)	
	Feminino	43,4 (10)	56,5 (13)	
IPAQ				0,732 §
	Baixo	4,4 (1)	30,4 (7)	
	Médio	65,2 (15)	60,9 (14)	
	Alto	30,4 (7)	8,7 (2)	
Fumante				0,108 §
	Não	100 (23)	73,9 (17)	
	Sim	0 (0)	26,1 (6)	
Classificação do IMC				0,571 §
	Abaixo do peso (<18,5kg/m^2^)	0 (0)	0 (0)	
	Peso normal (18,5>24,9kg/m^2^)	0 (0)	0 (0)	
	Sobrepeso (25>29,9kg/m^2^)	69,6 (16)	69,6 (16)	
	Obesidade (≥30kg/m^2^)	30,4 (7)	30,4 (7)	

Resultados expressos por mediana e intervalo interquartil (Q1 e Q3).

* teste de Mann-Whitney;

† teste *t* de Student;

‡ teste χ^2^de Person;

§ teste exato de Fisher.

GC: Grupo Controle; GE: Grupo Experimental; IMC: índice de massa corporal; FC: frequência cardíaca; PAS: pressão arterial sistólica; PAD: pressão arterial diastólica; IPAQ: *International Physical Activity Questionnaire* .

### Características da qualidade do ar

Em relação à caracterização do meio ambiente externo, de acordo com os valores de referência padrão nos municípios de Cubatão e Peruíbe, todos os itens foram estatisticamente significativos, com valores maiores no Grupo Experimental quando comparado com o Grupo Controle, como visto na tabela 2 .

**Tabela 2:** Comparação das características do meio ambiente externo (valor de referência) nos municípios de Cubatão e Peruíbe

Variáveis		Média/mediana	IC/IQR		Valor de p
CO_2_, ppm					<0,001 *
	GC	454,5	450,7	458,2	
	GE	479,1	473,1	485,1	
Temperatura, °C					<0,001 *
	GC	25,3	24,0	26,6	
	GE	20,2	19,5	20,9	
Umidade, %					0,003 †
	GC	46,4	44,9	46,7	
	GE	77,6	75,1	78,4	
MP 0,3 (x10^6^)					0,001 †
	GC	59,5	59,5	62,7	
	GE	214,7	211,4	216,8	
MP 0,5 (x10^6^)					0,001 †
	GC	4,9	4,7	5,4	
	GE	92,3	89,5	93,2	
MP 1,0 (x10^6^)					0,001 †
	GC	1,1	1,0	1,1	
	GE	11,6	10,2	12,0	
MP 3,0 (x10^6^)					0,001 *
	GC	0,9	0,9	0,9	
	GE	2,3	1,8	2,4	

* dados paramétricos (média e IC);

† dados não paramétricos (mediana e IQR).

IC: intervalo de confiança; IQR: intervalo interquartil (Q1 e Q3); CO_2_: dióxido de carbono; GC: Grupo Controle; GE: Grupo Experimental; MP: material particulado.

No ambiente interno da escola (sala de aula), apenas os itens dióxido de carbono e temperatura foram significantes, com valores maiores no Grupo Experimental quando comparado ao Grupo Controle ( Tabela 3 ).

**Tabela 3:** Comparação das características do ambiente interno da escola, de acordo com variáveis numéricas nos municípios de Cubatão e Peruíbe

Variáveis		Mediana	IQR (Q1 – Q3)		Valor de p
CO_2_, ppm					<0,001
	GC	528,0	520,7	542,2	
	GE	569,0	546,0	683,0	
Temperatura, ° C					<0,001
	GC	18,0	18,0	19,0	
	GE	19,0	19,0	20,0	
Umidade, %					0,409
	GC	73,9	72,2	75,6	
	GE	85,6	69,6	87,3	
MP 0,3 (x10^6^)					0,724
	GC	129,6	59,7	136,9	
	GE	237,3	32,2	252,6	
MP 0,5 (x10^6^)					0,852
	GC	7,9	4,1	8,6	
	GE	79,5	1,9	92,1	
MP 1,0 (x10^6^)					0,852
	GC	1,1	1,0	1,2	
	GE	8,1	0,3	9,7	
MP 3,0 (x10^6^)					0,851
	GC	0,8	0,8	0,9	
	GE	2,0	0,2	2,3	

teste de Mann-Whitney.

IQR: intervalo interquartil; CO_2_: dióxido de carbono; GC: Grupo Controle; GE: Grupo Experimental; MP: material particulado.

Em relação à caracterização do ambiente externo da escola (pátio da escola), os itens dióxido de carbono e umidade mostraram-se significantes, com valores maiores no Grupo Experimental quando comparado ao Grupo Controle ( Tabela 4 ).

**Tabela 4:** Comparação das características do meio ambiente externo (pátio da aula) da escola, de acordo com variáveis numéricas nos municípios de Cubatão e Peruíbe

Variáveis		Média/mediana	IC/IQR		Valor de p
CO_2_, ppm					0,005 *
	GC	471,0	465,0	476,5	
	GE	539,0	466,0	554,0	
Temperatura, ° C					0,295 *
	GC	17,0	16,0	17,0	
	GE	17,0	16,0	17,0	
Umidade, %					0,001 †
	GC	82,9	80,9	84,8	
	GE	88,4	85,6	91,2	
MP 0,3 (x10^6^)					0,221 *
	GC	127,2	66,1	161,8	
	GE	101,9	46,7	220,7	
MP 0,5 (x10^6^)					0,645 *
	GC	7,7	5,0	12,1	
	GE	8,5	2,6	111,7	
MP 1,0 (x10^6^)					0,701 *
	GC	1,1	1,0	1,5	
	GE	0,5	0,4	15,5	
MP 3,0 (x10^6^)					0,352 *
	GC	0,8	0,8	0,9	
	GE	0,5	0,3	3,8	

* dados não paramétricos (mediana e IQR);

† dados paramétricos (média e IC).

IC: intervalo de confiança; IQR: intervalo interquartil; CO_2_: dióxido de carbono; GC: Grupo Controle; GE: Grupo Experimental; MP: material particulado.

### Variabilidade da frequência cardíaca

Os itens SDNN, SD2, RRTri e TINN, que representaram a modulação da VFC geral, apresentaram significância após estatística analítica ( Tabela 5 ). De modo geral, ocorreu diminuição dessas variáveis no Grupo Experimental em comparação ao Grupo Controle.

**Tabela 5:** Índices lineares, domínio do tempo e da frequência, e geométricos da variabilidade da frequência cardíaca dos grupos selecionados

Índices da VFC	GC	GE	Valor de p
	(n=23)	(n=23)	
	Média (IC)	Média (IC)	
SDNN, ms	48,2 (43,2-53,1)	38,7 (33,5-43,8)	0,012 *
rMSSD, ms	27,5 (23,5-31,4)	22,6 (19,3-25,8)	0,061 *
pNN50, %	7,9 (4,4-11,4)	4,7 (2,9-6,6)	0,079 †
LFms^2^	738,1 (601,8-874,3)	541,8 (403-680,5)	0,054 *
LFnu	73,8 (69-78,5)	71,6 (67,1-76)	0,492 *
HFms^2^	254,8 (183,9-325,6)	208 (145,2-270,7)	0,441 ‡
HFnu	26 (21,2-30,7)	28,3 (23,8-32,7)	0,489 *
LF/HF	3,6 (2,7-4,5)	3,1 (2,3-3,9)	0,460 ‡
SD1, ms	19,5 (16,7-22,2)	16 (13,7-18,2)	0,062 *
SD2, ms	65,1 (58,2-71,9)	52,1 (44,9-59,2)	0,013 *
SD1/SD2, ms	0,304 (0,267-0,340)	0,317 (0,279-0,354)	0,625 *
RRTri, ms	12,9 (11,6-14,1)	10,3 (8,9-11,6)	0,009 *
TINN, ms	214,3 (193,8-234,7)	172,8 (150,5-195)	0,010 *

* teste *t* de Student sem ajuste;

† teste *t* de Student após ajuste com raiz quadrada;

‡ teste *t* de Student após ajuste com transformada logaritmica.

VFC: variabilidade da frequência cardíaca; GC: Grupo Controle; GE: Grupo Experimental; IC: intervalo de confiança; SDNN: desvio padrão de todos os intervalos RR normais; rMSSD: raiz quadrada da média do quadrado das diferenças entre intervalos RR normais adjacentes; pNN50: percentagem das diferenças sucessivas do intervalo RR cujo valor absoluto excedeu 50ms; LF: componente de baixa frequência; nu: unidades normalizadas; HF: componente de alta frequência; SD1: desvio padrão da variabilidade instantânea batimento a batimento em curto prazo; SD2: desvio padrão a longo prazo dos intervalos RR contínuos; relação SD1/SD2: dispersão de pontos perpendiculares à linha de dispersão identidade de pontos ao longo da identidade da linha; RRTri: índice triangular; TINN: interpolação triangular de histograma de intervalos NN.

## DISCUSSÃO

Os resultados do presente estudo mostraram que o Grupo Experimental composto por indivíduos com excesso de peso expostos à poluição atmosférica apresentou diminuição nos valores dos índices SDNN, SD2, RRTri e TINN (que correspondem à modulação global da VFC) quando comparado ao Grupo Controle, sugerindo que, nestes indivíduos, a VFC encontra-se reduzida.

A literatura sugere padronização dos valores dos índices da VFC entre os indivíduos supostamente saudáveis. Uma tentativa foi realizada no estudo de Sammito et al.,^(^^17^^)^que registraram os valores da VFC de longa duração (24 horas) com a utilização do eletrocardiograma (ECG) em adultos saudáveis. Como resultado, os autores observaram diferenças nos valores entre as diferentes idades e sexo. Entre as variáveis do domínio do tempo, os valores da média das variáveis SDNN e rMSSD para os homens de 30 a 40 anos foram 48,98 e 40,71 milissegundos, respectivamente. Em relação à variável pNN50 para a mesma faixa etária, foi 13,23%. Entre as mulheres de 30 a 40 anos, os valores das médias foram menores: 42,39 para SDNN, 36,50 para rMSSD e 11,43% para pNN50. No mesmo estudo,^(^^17^^)^os índices do domínio da frequência foram também pesquisados. Os valores da média das variáveis LF e HF em unidades normalizadas (LFnu e HFnu) para os homens de 30 a 40 anos foram 75,46 e 24,54, respectivamente. Entre os homens desta idade, a LF/HF foi 3,08. As mulheres de 30 a 40 anos apresentaram médias de 67,81 para LFnu, 32,19 para HFnu e 2,11 para LF/HF.^(^^17^^)^

Outro estudo, realizado por Sandercock et al.,^(^^18^^)^também mensurou os valores da VFC em adultos saudáveis, porém com gravação de curta duração (5 a 10 minutos) com a utilização do cardiofrequencímetro da marca Polar. Os valores dos índices da VFC variaram de acordo com a intensidade da prática física. Como resultado, os valores encontrados entre homens e mulheres, com IMC de 22 a 23kg/m^2^, e média de idade de 23 anos (variando entre 18 e 33 anos), com baixo, moderado e alto nível de atividade física, foram 55, 75 e 64 milissegundos para SDNN; 56, 78 e 60 milissegundos para rMSSD; 58, 57 e 65 para LFnu; 42, 43 e 35 para HFnu; 6,95, 7,26 e 7,31ms^2^para LFms^2^; 6,59, 6,95 e 6,60 milissegundos ao quadrado para HFms^2^; e 2,0, 1,7 e 2,6 para LF/HF, respectivamente.^(^^18^^)^

De modo geral, os valores dos índices SDNN, rMSSD e pNN50 dos estudos citados (adultos saudáveis) apresentaram-se mais elevados quando comparados com os valores do Grupo Controle e Grupo Experimental desta pesquisa; já os valores dos índices do domínio da frequência mostraram-se variados, de acordo com o sexo e o nível de atividade física, sugerindo que o ganho de peso pode ser associado a uma diminuição da VFC, indicando descontrole autonômico.^(^^19^^)^Esses achados corroboram outros estudos citados na literatura, que compararam indivíduos com sobrepeso e peso normal.^(^^20^^–^^26^^)^

Uma das possíveis explicações sobre essas alterações na VFC é a ativação do barorreflexo. Estudo^(^^27^^)^que testou a hipótese de que a sensibilidade do barorreflexo (SBR), avaliada pela medida indireta da pressão aórtica, está relacionada com a obesidade, selecionou um grupo de 30 mulheres com IMC de 30kg/m^2^e um grupo de 30 controles com IMC de 25kg/m^2^. A SBR foi estimada pela atividade do SNA cardíaco pela VFC. Deste modo, o SBR foi menor em mulheres obesas; já os valores de HF e LF foram maiores nos participantes magros do que nos obesos (1.079,2 *versus* 224,1ms^2^, e 411,8 *versus* 235,8ms^2^, respectivamente) e o valor de LF/HF foi maior nos indivíduos obesos (0,82 *versus* 0,57). Concluiu-se, então, que o SBR é severamente reduzido em indivíduos obesos. O IMC, a idade e a atividade do sistema nervoso parassimpático são os principais determinantes da SBR. O comportamento do barorreflexo é de relevância clínica, pois, quando atenuado, representa fator prognóstico negativo nas DCV, comuns na obesidade.^(^^27^^)^

Quando os indivíduos com excesso de peso estão expostos a poluentes atmosféricos, os valores da VFC mostram-se ainda mais alterados. Em geral, a literatura aponta relação inversa significativa entre a exposição a poluentes atmosféricos, principalmente ao MP, e diminuições nos índices SDNN, rMSSD, HF, até mesmo no LF para indivíduos com excesso de peso expostos a altos níveis dessas partículas atmosféricas.^(^^28^^–^^34^^)^Contudo, no resultado do presente estudo, apenas o índice SDNN do domínio do tempo, e os índices SD2, RRTri e TINN dos índices geométricos apresentaram redução significante em comparação ao Grupo Controle, sugerindo alteração na variabilidade global. Os outros índices, como rMSSD, pNN50, LFms^2^e SD1, também demonstraram valores menores em relação ao Grupo Controle, embora não estatisticamente significativos, variando entre 0,054 e 0,07.

### Limitações

O presente estudo apresenta algumas limitações que devem ser consideradas. Algumas medições não puderam ser realizadas, como, por exemplo, a utilização da razão cintura-quadril, para análise do risco de desenvolvimento de DCV, que possibilitaria a melhor divisão entre os grupos. Outra restrição foi a perda amostral – cerca de 20 exclusões (30,3% de perda amostral) – em razão, principalmente, de erros na VFC. Assim, recomenda-se acréscimo de 31% no cálculo amostral em futuras pesquisas. Ademais, não foi realizada a coleta de outros poluentes, a qual permitiria demonstrar, com mais precisão, a exposição dos diferentes poluentes nos indivíduos estudados.

## CONCLUSÃO

Indivíduos com excesso de peso expostos à poluição atmosférica apresentaram menor variabilidade da frequência cardíaca global quando comparado ao Grupo Controle. Além disso, as modulações parassimpática e simpática mostraram-se com tendência de queda, embora não significativa.

As descobertas desta pesquisa sugerem que os indivíduos com excesso de peso expostos à poluição atmosférica apresentaram desequilíbrio autonômico e, assim, uma maior suscetibilidade ao desenvolvimento de doenças cardiovasculares.
